# Evaluating the safety profile of the CoronaVac in adult and older adult populations: A phase IV prospective observational study in Brazil

**DOI:** 10.1371/journal.pgph.0004069

**Published:** 2025-02-25

**Authors:** Vanessa Infante, Monica Akissue de Camargo Teixeira Cintra, Eder Gatti Fernandes, Ana Paula Loch, Lucas Ragiotto, Patrícia Emília Braga, Maria da Graça Salomão, Maria Beatriz Bastos Lucchesi, Mayra Martho Moura de Oliveira, Vera Lúcia Gattás, Anderson Soares da Silva, Paulo José Fortes Villas Boas, Marta Heloisa Lopes, José Moreira, Fernanda Castro Boulos, CFV-01-IB study group

**Affiliations:** 1 Clinical Trials and Pharmacovigilance Center, Instituto Butantan, São Paulo, Brazil; 2 Centro de Saúde Escola da Faculdade de Medicina de Ribeirão Preto da Universidade de São Paulo (HCFMRP-USP) Dr. Joel Domingos Machado, São Paulo, Brazil; 3 Centro de Saúde Escola da Faculdade de Medicina de Botucatu—Unesp, São Paulo, Brazil; 4 Centro de Referência de Imunobiológicos Especiais Hospital das Clínicas da Faculdade de Medicina da Universidade de São Paulo (CRIE-HCFMUSP), São Paulo, Brazil; Aarhus University: Aarhus Universitet, DENMARK

## Abstract

This Phase IV prospective observational study aimed to evaluate the frequency of solicited and unsolicited adverse reactions within seven days following the administration of each dose of CoronaVac (14-day interval) by age group (18–59 years and ≥60 years). Participants (n = 538; 487 adults and 51 older adults) were enrolled from three public health centers in São Paulo, Brazil from May 2021 to January 2022. The study involved a two-dose vaccination regimen administered 14 days apart. Solicited and unsolicited adverse reactions (ARs) were assessed within seven days after each dose, and medically attended adverse events following immunization (AEFI) were monitored for 42 days. Safety data were collected through participant diary cards, telephone follow-ups, and on-site visits. Among adults, the most frequently reported local AR after the first and second doses was pain (256 [52.6%] and 129 [29.5%], respectively), while the most common systemic AR was headache (158 [34.5%] and 51 [11.6%], respectively). Most local and systemic solicited ARs were of Grade 1 or 2 severity, with ARs being more prevalent in adults following the first dose. One serious adverse event related to the vaccine was reported in adults, with no fatalities. Nine adult participants experienced adverse events of special interest, including five cases of COVID-19. These findings support the overall safety profile of CoronaVac in adults and older adult individuals, with adverse events being generally mild and self-limited.

## Introduction

The rapid development and subsequent market authorization of SARS-CoV-2 vaccines in response to the COVID-19 pandemic have highlighted the critical role of pharmacovigilance in managing the large volume of reported adverse events following immunization (AEFI). This process is essential for ensuring the safety and effectiveness of global vaccination campaigns [1].

Instituto Butantan (IB), a prominent Brazilian public biomedical research and manufacturing institution, has been instrumental in COVID-19 vaccine governance in Brazil. In 2020, IB initiated a phase 3 clinical trial to assess the efficacy and safety of CoronaVac among frontline healthcare professionals in Brazil [2]. On January 17th, CoronaVac was granted Emergency Use Authorization (EUA) by the National Health Surveillance Agency (Anvisa).

Despite the large sample size in phase trials, very rare adverse events may remain undetected, as these trials typically rely on spontaneous reporting. Post-marketing safety surveillance is essential to ensure the ongoing safety of the vaccine and support equitable vaccine distribution. In line with Anvisa’s recommendations, IB conducted a post-market approval safety evaluation to characterize AEFI associated with CoronaVac in adults and older adults during Brazil’s vaccination campaign.

## Methods

### Study design

#### Ethics statement.

This phase IV prospective observational study monitored the safety of the CoronaVac, COVID-19 adsorbed vaccine, following the administration of at least one dose in adults (18–59 years) and older adults (≥ 60 years). The study was registered on ClinicalTrials.gov (NCT04845048) and conducted between May 2021 and January 2022 at three public health centers in São Paulo state, Brazil. Ethical approval was obtained from the University of São Paulo’s coordinating study site (CAAE - 44291021.7.1001.0068, protocol number: 4.684.670) and collaborating study sites. The study started after ANVISA issued the EUA for CoronaVac on January 17th, 2021. Ethical approval was obtained from the coordinating study site (University of São Paulo) and the collaborating study sites (Centro de Referência de Imunobiológicos Especiais/Hospital das Clínicas da Faculdade de Medicina da Universidade de São Paulo (CRIE-HCFMUSP), Centro de Saúde Escola da Faculdade de Medicina de Ribeirão Preto da Universidade de São Paulo (HCFMRP-USP), and Centro de Saúde Escola da Faculdade de Medicina de Botucatu—Unesp). Written informed consent was obtained from all participants, and the study followed the Strengthening the Reporting of Observational Studies in Epidemiology (STROBE) guidelines for reporting observational studies (S1 Checklist).

### Subjects

Inclusion criteria required participants to be at least 18 years old and eligible for COVID-19 vaccination as determined by Brazil’s National Immunization Program [3]. Initial recommendations prioritized vulnerable populations, including, healthcare professionals, older adults, and immunocompromised individuals. The exclusion criteria included a history of allergic reactions or anaphylaxis to previous immunizations, allergy to any CoronaVac component, fever (axillary temperature ≥ 37.8 °C) within 72 hours prior to vaccination, or unavailability for follow-up during the study period. Prior COVID-19 infection was not an exclusion criterion.

### Primary and secondary endpoints

The primary endpoint was the frequency of solicited and unsolicited adverse reactions (ARs) within seven days post-administration of each CoronaVac dose, stratified by age group by age group. Secondary endpoints included the frequency of medically attended AEFI within 42 days post-administration of each dose; AR within 30 minutes post-administration of each dose; serious adverse event (SAE) within 42 days post-administration of each dose of the vaccine, and adverse event of special interest (AESI) within 42 days post-administration of each dose of the vaccine. Exploratory endpoints assessed the adverse events (AE) among pregnant women and their exposed infants.

### Study procedures for active surveillance

During the first visit (V1) participants received the first dose of the vaccine, were observed for 30 minutes, and were given a diary card to record ARs over seven days. The second visit (V2) occurred at least 14 days later, during which participants were evaluated, and data from the diary card were collected. In this visit the eligible criteria were evaluated, and the diary card and information on concomitant medication administration were collected. A second vaccine dose was then administered, followed by a 30-minute observation period. After that, the participant received the second vaccine dose and remained under observation for 30 minutes. In addition to on-site visits, seven scheduled phone contacts were conducted to monitor ARs and collect safety data. The phone contacts C1 (V1 + 3–5 days), C2 (V1 + 7–9 days), C3 (V2 + 3–5 days), C4 (V2 + 7–9 days), C5 (V2 + 14–16 days), C6 (V2 + 42–45 days) and C7—end of study (V2 + 60–63 days) were scheduled based on vaccination date. Demographic characteristics (sex, age and race), medical history, concomitant medication, and information about pregnancies were collected in all visits/contact. Furthermore, in V2 and in all phone contacts, the occurrence of adverse events and adverse events that required medical care and/or hospitalization were evaluated.

### Safety assessment

An adverse event was defined as any untoward medical occurrence in a participant after the administration of the vaccine which does not necessarily have a causal relationship with the intervention. All ARs were encoded and categorized using the Medical Dictionary for Regulatory Activities (MedDRA, version 24.0) [4]. Serious Adverse Events (SAEs) were defined as events leading to outcomes such as death, a life-threatening condition, hospitalization, prolonged current hospitalization, significant or persistent incapacitation, congenital anomaly, any suspected transmission of an infectious agent through a medicinal product, or any clinically significant adverse event. ARs were considered vaccine-related based on the adapted classification from the Uppsala Monitoring Centre (UMC) of the World Health Organization (WHO) [5–9] (S1 Appendix and S2 Appendix). AESIs were predefined, using the Brighton Collaboration’s list for COVID-19 vaccines [10].

The severity of an AE or AR was classified from grade one to four according to the Toxicity Grading Scale for Healthy Adult and Adolescent Volunteers Enrolled in Preventive Vaccine Clinical Trials of the US Food and Drug Administration (FDA) [8].

The definitions and classifications of AR followed the recommendations stipulated by the Good Clinical Practices (GCP) [9], the Guideline on Clinical Safety Data Management from the International Conference on Harmonization (ICH) [6], and the Brighton Collaboration’s guidance on safety data collection for COVID-19 vaccine safety [10]. Whenever case definitions were not provided by the Brighton Collaboration sources, the Common Terminology Criteria for Adverse Events (CTCAE) issued by the US Department of Health and Human Services [11] were utilized as references.

### Study vaccine

The study vaccine, CoronaVac, is an inactivated whole-virion vaccine formulated with aluminum hydroxide as an adjuvant, prepared with a novel coronavirus strain (CZ02 strain) inoculated in African green monkey kidney cells (Vero cells). The inactivation process involved adding β-propiolactone to the virus harvest fluid at a ratio of 1:4000 and inactivating at 2–8 °C for 12–24 h. A single dose of the vaccine contains 3 μg of SARS-CoV-2 virion in a 0.5 mL aqueous suspension for injection with 0.45 mg/mL of aluminum. The vaccine was administered intramuscularly in the deltoid muscle in two doses, 14 days apart. The vaccine is free of preservatives and the final packaging includes a single-dose vial with an extractable volume of 0.5 mL, a vial with two doses of 0.5 mL each, or a multi-dose vial containing 5 mL, with each vaccine dose corresponding to 0. 5 mL.

### Sample size

The sample size was estimated based on the recommendations of the Guide to Clinical Evaluations of New Vaccines (EMEA/CHMP/VWP/164653/2005) [12], suggesting that in pre-authorization studies it is possible to determine the frequency of uncommon local and systemic adverse events, i.e., events that occur at least once between 100 and 1000 vaccinated people. Thus, a sample size of 900 vaccinated participants (600 adults and 300 older adults) was targeted for active surveillance of solicited and unsolicited AEFI.

This sample size was based on the formula presented below, assuming the observation of an AE at an established minimum frequency equal to M (maximum risk). N = ln(α)/ln(1-M) Type I error parameter (α) adopted was 5% (two-tailed) and N for older adults = 300, to achieve AE frequency detection of approximately 1% (maximum risk of 1 to 100), whereas N for adults = 600 achieves AE frequency at a maximum risk of 1 in 200.

### Statistical analysis

Descriptive statistics were used to summarize the occurrence of AE and AR by age group and dose [8]. Results were presented as frequency for categorical variables (absolute and percentage values) and as dispersion measures for continuous variables. Missing data were handled with list-wise deletion. The statistical analyses were conducted using Stata (Stata-Corp LP, College Station, Texas USA, version 13.0, for Windows).

## Results

### Participants

**Fig 1:**
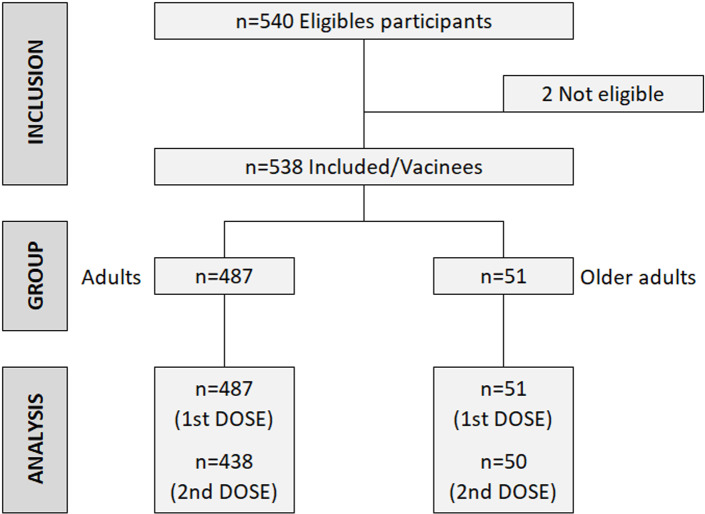
Study flowchart: screening, enrollment, and vaccination.

Fig 1 shows the study participants’ flowchart, by age groups. The study enrolled 538 participants, comprising 487 adults (90.5%) and 51 older adults (9.5%). The median age for adults was 30.5 years (IQR) for adults and 65.3 years for older adults (Table 1). Most participants were females (57.1%). Thirty-nine participants did not receive the second vaccine dose due to loss of follow-up.

**Table 1:** Demographic characteristics of all participants by study group.

Variables	Adults 18–59 years old	Older adults ≥60 years old	Total
	(n = 487)	(n = 51)	(N = 538)
**Age (years)**			
Median (Q1–Q3)	30.5 (25.5–41.6)	65.3 (64.2–68.9)	32 (26.0–46.0)
**Sex, n (%)**			
Female	276 (56.7)	31 (60.8)	307 (57.1)
Male	211 (43.3)	20 (39.2)	231 (42.9)
**Ethnicity, n (%)**			
White	289 (59.3)	30 (58.8)	319 (59.3)
Black	50 (10.3)	8 (15.7)	58 (10.8)
Multiracial	125 (25.7)	7 (13.7)	132 (24.5)
Indigenous	1 (0.2)	3 (5.9)	4 (0.7)
Asian	11 (2.3)	3 (5.9)	14 (2.6)
Other	11 (2.3)	0 (0.0)	11 (2.0)
**Clinical Sites, n (%)**			
Site 01	216 (44.4)	35 (68.6)	251 (46.7)
Site 02	180 (37.0)	16 (31.4)	196 (36.4)
Site 03	91 (18.7)	0 (0.0)	91 (16.9)

Q1: first quartile; Q3: third quartile.

### Primary endpoint

Among all participants, 73.8% experienced at least one AR after the first dose, with 76.4% in the adults and 45.1% in older adults. After the second dose, ARs were less frequent in both groups (Table 2).

**Table 2:** Frequency of solicited and unsolicited Adverse Events and Adverse Reactions after CoronaVac vaccination (first and second dose) by study group.

Study group	1st Dose				2nd Dose			
	AE n	Participants with AE n/n_total _(%)	AE per participant		AE n	Participants with AE n/n_total _(%)	AE per participant	
			Median	(Q_1_-Q_3_)			Median	(Q_1_–Q_3_)
Adults (18–59 years)	1569	392/487 (80.5)	3	(2–5)	554	218/438 (49.8)	2	(1–3)
Older adults (≥60 years)	136	30/51 (58.8)	3	(1–6)	94	28/50 (56.0)	3	(1–5)
**Total**	1705	422/538 (78.4)	3	(2–5)	648	246/488 (50.4)	2	(1–3)
**Study group**	**AR n**	**Participants with AR n/n**_**total **_ **(%)**	**AR per participant**		**AR n**	**Participants with AR n/n**_**total **_ **(%)**	**AR per participant**	
			**Median**	**(Q**_**1**_ **–Q**_**3**_ **)**			**Median**	**(Q**_**1**_ **–Q**_**3**_ **)**
Adults (18–59 years)	1288	374/487 (76.8)	3	(1–5)	438	194/438 (44.3)	2	(1–3)
Older adults (≥60 years)	84	23/51 (45.1)	3	(1–4)	53	22/50 (44.0)	1	(1–4)
**Total**	1372	397/538 (73.8)	3	(1–5)	491	216/488 (44.3)	2	(1–3)

Q1: first quartile; Q3: third quartile.

AE: Adverse Event.

AR: Adverse Reaction.

For adults, 76.0% had at least one solicited AR after the first dose and 42.5% after the second dose, within the 7-day post-vaccination period (Table 3). The most common local AR after both doses was pain at the injection site (52.6% after the first dose, 29.5% for the second dose, and the most frequent systemic AR was headache (34.5% for the first dose, 11.6% after the second dose). Most local and systemic solicited ARs were of Grade 1 or 2 severity (S1 Table). Thirty-five solicited AR (3.0%) were classified as Grade 3 after the first dose, while 10 (2.6%) after the second dose. Only three ARs were classified as Grade 4 (1 headache, 1 fatigue and 1 myalgia), all following the first dose.

**Table 3:** Frequency of solicited adverse reactions (AR) 7 days post-vaccination in adults (18–59 years).

Solicited Adverse Reactions (AR)	1st Dose			2nd Dose		
	AR in Adults (n = 487)		AR Total	AR in Adults (n = 438)		AR Total
	n of participants	%	n of events	n of participants	%	n of events
**Local**						
Pain	256	52.6	263	129	29.5	132
Erythema	9	1.8	9	10	2.3	11
Swelling	15	3.1	15	3	0.7	4
Induration	10	2.1	11	3	0.7	3
Pruritus	15	3.1	15	4	0.9	4
Tenderness	166	34.1	169	59	13.5	59
**Systemic**						
Decreased appetite	20	4.1	20	6	1.4	7
Arthralgia	40	8.2	44	11	2.5	11
Chills	35	7.2	37	9	2.1	9
Headache	168	34.5	191	51	11.6	54
Diarrhea	36	7.4	38	14	3.2	15
Rash	8	1.6	8	1	0.2	1
Fatigue	107	22.0	127	23	5.3	25
Fever (Pyrexia)	14	2.9	14	5	1.1	5
Hypersensitivity (Allergic Reaction)	2	0.4	2	0	0.0	0
Myalgia	69	14.2	72	21	4.8	22
Nausea	39	8.0	43	16	3.7	17
Pruritus Outside the Administration Site	32	6.6	33	5	1.1	5
Cough	38	7.8	39	5	1.1	5
Vomiting	6	1.2	7	2	0.5	2
**Total solicited**	370	76.0	1157	186	42.5	391

For older adults, 43.1% experienced at least one solicited AR after the first dose, and 44.0% after the second dose, within a 7-day post-vaccination period (Table 4). The most common local AR was pain at the administration site (17.6% and 22.0% after the first and second dose, respectively) and the most frequent systemic AR was headache (9 [17.7%] and 10 [20.0%] after the first and second dose, respectively). Most local and systemic solicited AR were classified as Grade 1 or Grade 2 (S2 Table). Only one episode of myalgia was classified as Grade 3, and no local and systemic solicited AR were classified as Grade 4.

**Table 4:** Frequency of solicited adverse reactions (AR) 7 days post-vaccination in older adults (≥60 years).

Solicited Adverse Reactions (AR)	1st Dose			2nd Dose		
	AR in Older adults (n = 51)		AR Total	AR in Older adults (n = 50)		AR Total
	n of participants	%	n of events	n of participants	%	n of events
**Local**						
Pain	9	17.6	10	11	22,0	11
Swelling	0	0.0	0	1	2.0	1
Itching	1	2.0	1	2	4.0	2
Tenderness	8	15.7	8	1	2.0	1
**Systemic**						
Decreased Appetite	1	2.0	1	0	0,0	0
Arthralgia	7	13.7	9	4	8.0	4
Chills	3	5.9	4	4	8.0	6
Headache	9	17.6	9	10	20.0	12
Diarrhea	3	5.9	3	2	4.0	2
Rash	1	2.0	1	2	4.0	2
Fatigue	7	13.7	7	1	2.0	1
Fever (Pyrexia)	0	0.0	0	1	2.0	1
Myalgia	8	15.7	8	3	6.0	3
Nausea	2	3.9	2	2	4.0	2
Pruritus Outside the Administration Site	4	7.8	4	1	2.0	1
Cough	1	2.0	1	1	2.0	1
Vomiting	1	2.0	1	0	0.0	0
**Total solicited**	22	43.1	69	22	44.0	50

Frequency and severity of solicited and unsolicited AR within 7 days post-vaccination for adults and older adults are available in the Supplementary material (S2 Table).

### Secondary endpoint

Table 5 displays the frequency of adults and older adults experiencing solicited ARs within 30 minutes post each vaccination. The solicited AR were more common after the first dose among adults (26.5% vs 19.2%, first and second dose respectively). Solicited AR were less frequent among the older adults, with no noticeable difference between the first and the second dose (11.8% and 12.0%, respectively). The frequency and intensity of unsolicited AR within 30 minutes post-vaccination in adults and older adults can be found in the S3 Table.

**Table 5:** Frequency of Adverse Reactions (AR) within 30 minutes post vaccination in adults (18–59 years) and older adults (≥60 years).

Solicited Adverse Reactions (AR)	1st Dose			2nd Dose			1st Dose			2nd Dose		
	AR Adults (n = 487)		AR Total	AR Adults (n = 438)		AR Total	AR Older adults (n = 51)		AR Total	AR Older adults (n = 50)		AR Total
	n of participants	%	n of events	n of participants	%	n of events	n of participants	%	n of events	n of participants	%	n of events
**Local**												
Pain	85	17.5	85	52	11.9	52	4	7.8	4	6	12.0	6
Erythema	2	0.4	2	2	0.5	2	0	0.0	0	0	0.0	0
Induration	5	1.0	5	3	0.7	3	0	0.0	0	0	0.0	0
Itching	1	0.2	1	1	0.2	1	0	0.0	0	1	2.0	1
Tenderness	48	9.9	48	32	7.3	32	2	3.9	2	1	2.0	1
**Systemic**												
Arthralgia	0	0.0	0	1	0.2	1	0	0.0	0	1	2.0	1
Headache	9	1.8	9	4	0.9	4	1	2.0	1	0	0.0	0
Fatigue	3	0.6	3	3	0.7	3	0	0.0	0	0	0.0	0
Myalgia	3	0.6	3	1	0.2	1	0	0.0	0	0	0.0	0
Nausea	3	0.6	3	2	0.5	2	0	0.0	0	0	0.0	0
Cough	1	0.2	1	1	0.2	1	0	0.0	0	0	0.0	0
Vomiting	0	0.0	0	0	0.0	0	0	0.0	0	0	0.0	0
**Total solicited**	129	26.5	160	84	19.2	102	6	11.8	7	6	12.0	9

In the adult group, it was observed that 2.1% of participants who had at least one solicited medically attended AR within 7 days after the first vaccine dose and 0.2% after the second dose. The most common solicited AR that needed medical care was headache. Regarding unsolicited medically attended AR, 1.8% of participants had at least one after 42 days after the first vaccine dose and 0.2% after the second dose. The most common unsolicited medically attended AR was rhinorrhea. In the older adults, none solicited medically attended AR were observed after the first dose, for the second dose, one diarrhea and one arthralgia were observed. Regarding unsolicited medically attended AR, 42 days after first vaccination, one palpitation and one flu-like disease were observed (S4 Table).

All participants that presented an AR that required medical intervention or an unexpected SA had fully recovered, except one unexpected SAE (migratory polyarthralgia) that was assessed by the investigator and considered related to the vaccine and recovery with sequelae.

Nine adult participants experienced at least one AESI. These included five cases of COVID-19, two cases of arthritis, one case of tachycardia, and one case of acute renal failure. Only two AESI were considered related to the vaccine, one arthralgia and one tachycardia, both mild (S5 Table). Regarding COVID-19 infection, only two cases required hospitalization, but all recovered.

### Exploratory endpoints

Among two pregnant participants, seven non-serious ARs were reported, and all participants recovered. Detailed information about the AE in pregnant women and infants can be found in Supplementary material (S6 Table). Among the women who got pregnant during vaccination, one was 26 years old, black, with a history of cirrhosis and hypertension, in addition to having had a previous pregnancy with complications (Preeclampsia). She was vaccinated with a second vaccine dose on the 8th gestational week and developed preeclampsia with premature birth (34 weeks) and the child was small for that gestational age. The other participant was 21 years old, white, with no comorbidity and no previous pregnancy. She was vaccinated with a second vaccine dose on 7th gestational week and developed preeclampsia requiring an emergency c-section at 37 gestational weeks.

## Discussion

The expedited development and EUA of COVID-19 vaccines were instrumental in mitigating the global impact of the SARS-CoV-2 pandemic, particularly during its acute phase [13]. Among these vaccines, CoronaVac, developed by Sinovac Biotech and transferred to Instituto Butantan (IB), has been widely used in numerous countries. However, as with all COVID-19 vaccines authorized during the public health emergency, safety data for CoronaVac were initially limited, underscoring the need for rigorous post-vaccination real-world safety data.

Recognizing this knowledge gap, Anvisa commissioned a comprehensive post-authorization safety study on CoronaVac in adults and older adults. The primary objective was to characterize AEFI, especially among populations that were less exposed to the virus than healthcare professionals [2]. The real-world nature of this study provided the opportunity to evaluate the vaccine’s safety profile in a broader and more diverse population than those enrolled in clinical trials.

Our findings demonstrate that the most commonly reported ARs were pain at the injection site, followed by headache and myalgia in both adults and older adults. Most ARs were mild to moderate in severity, with 83.15% classified as Grade 1 and 13.5% as Grade 2, consistent with previous studies evaluating this vaccine [6,13–20] The majority of solicited ARs resolved within 24 hours, differing from the results of Xia et al. [21] and Zhang et al. [22], in which AR lasted up to 72h post-vaccination in healthy adults. Previous studies on inactivated virus vaccines, such as those by Ling et al. [23] and Chen et al. [24], similarly identified pain, swelling, and fever as the most common local reactions, with headache and malaise being the most significant systemic reactions. Our result also aligns with data from Indonesia, which indicated a higher frequency of AEFI in female participants. Regarding the most frequent local and systemic adverse reactions by the inactivated virus COVID-19 vaccine, Ling et al. [23] reported pain, swelling, and fever at the injection site, whereas Chen et al. [24] reported pain, swelling and redness at the injection site. Results from randomized trials in Indonesia have listed headache and malaise as the most significant systemic AEFI [25]. Indonesian results pointed out gender-related AEFI after CoronaVac immunization in which females presented a higher frequency of AEFI than male participants. In terms of SAE, we observed one case of migratory polyarthritis related to the vaccine, a finding not reported in other published studies [17,18,20–24,26,27]. Tanriover et al. [19], in a phase III study conducted in Turkey, identified two vaccine-related SAEs as seizure and an allergic reaction.

Whereas some AESI were observed, only two (arthritis and tachycardia) were deemed related to the vaccine, both of which were mild and resolved without complications. The Global Vaccine Data Network (GVDN) cohort study [28], which evaluated AESI across 99 million vaccines, reported a higher risk of Guillain-Barré Syndrome, transverse myelitis, and acute disseminated encephalomyelitis (ADEM) following the ChAdOx1 vaccine and ADEM and seizure following mRNA-1273 vaccine. No such events were observed in our study.

Regarding vaccination in pregnant women, our study observed a very small number vaccinated pregnant women (n = 2), both of them with complications. However, a retrospective cohort study conducted in Rio de Janeiro city between May and October 2021, which included over 17,000 live births, found no significant increase in adverse outcomes such as premature birth, low birth weight, or neonatal death following vaccination [26].

Brazilian national passive surveillance data reported a 3.0% rate of SAE following CoronaVac [14], whereas our study identified a rate of 0.6%. Unlike passive pharmacovigilance, which relies on voluntary spontaneous reporting of AE, active pharmacovigilance facilitates the procurement of more detailed safety information. Passive surveillance is more sensitive to SAE, but the data quality is weak [15,16]. A key strength of our study was the use of active surveillance through follow-up phone calls after each dose, enabling the capture of more detailed information on mild and moderate events, which are typically underreported in passive systems. Furthermore, the low dropout rate in our study reinforces the vaccine’s good tolerability.

Our study has limitations. The sample size did not reach the originally calculated target due to the public vaccination campaign commencing three months earlier than protocol approval, during which other vaccines were also administered. Consequently, the reduced sample size impacted the study’s power to detect adverse events, particularly in older adults. Due to this difficulty in including unvaccinated subjects, and therefore, the reduction in sample size, the minimum AE detection power for adults was M = 1/165≈0.6% whereas for older adults was M = 1/18≈5.7%. Additionally, the demographic distribution, with a larger proportion of younger adults, may have introduced bias, potentially explaining the higher reactogenicity observed in adults compared to older individuals. Our study was not designed to assess COVID-19 cases, and while severe cases were captured as SAEs, milder cases could have been underreported due to limited virologic testing.

Following the EUA extension of CoronaVac’s EUA to children and adolescents in January 2022, the study protocol was amended to include these groups, allowing safety data collection in younger populations. Despite the sample size limitations, our findings are consistent with other studies, suggesting that CoronaVac is well tolerated with a relatively lower frequency of AR across age groups [17,18].

In conclusion, our study adds to the growing body of evidence supporting the safety of CoronaVac. It provides clinicians with crucial information for patient counselling regarding potential side effects and offers policymakers valuable data to guide vaccination strategies. Our findings help consolidate the safety profile of CoronaVac within Brazil’s COVID-19 vaccine response.

## Supporting information

S1 Checklist STROBE statement. (DOCX)

S1 Appendix Severity classification. (DOCX)

S2 Appendix Causal relationship classification. (DOCX)

S1 Table Frequency and severity of adverse reactions, solicited (local and systemic) and unsolicited, occurring up to 7 days after administration of each vaccine dose in adults (18–59 years), according to severity. (DOCX)

S2 Table Frequency and severity of adverse reactions, solicited (local and systemic) and unsolicited, occurring up to 7 days after administration of each vaccine dose in older adults (≥60 years), according to severity. (DOCX)

S3 Table Frequency of adverse reactions, solicited (local and systemic) and unsolicited, occurring within 30 minutes after administration of each vaccine dose in a) adults (18–59 yeas) and b) older adults (≥60 years), according to severity. (DOCX)

S4 Table Frequency of solicited (local and systemic) and unsolicited adverse reactions which required medical attention, occurring at any time within 42 days after administration of each vaccine dose in a) adults (18–59 years) and b) older adults (≥60 years), according to severity. (DOCX)

S5 Table Serious adverse events (SAE) and adverse events of special interest (AESI) according to the administration dose regarding description (MedRA code), causality, severity, predictability and outcomes. (DOCX)

S6 Table Adverse Event (AE) details observed in pregnant women and newborn according to the administration dose regarding description code (MedRa), causality, severity, predictability and outcomes. (DOCX)
