# Supplementary material for: Evaluating the safety profile of the CoronaVac in adult and older adult populations: A phase IV prospective observational study in Brazil
Source: PLOS Glob Public Health. 2025 Feb 25;5(2):e0004069. doi: 10.1371/journal.pgph.0004069 (PMC12048030; doi:10.1371/journal.pgph.0004069)
Supplement: S1 Checklist — (DOCX) [file pgph.0004069.s001.docx]

S1. Checklist - STROBE Statement— list of items that should be included in reports of observational studies

|  | Item No. | Recommendation | Page  No. | Relevant text from manuscript |
| --- | --- | --- | --- | --- |
| **Title and abstract** | 1 | (*a*) Indicate the study’s design with a commonly used term in the title or the abstract | 1 | A Phase IV Prospective Observational study in Brazil |
|  |  | (*b*) Provide in the abstract an informative and balanced summary of what was done and what was found | 2 | Abstract |
| Introduction | | | |  |
| Background/rationale | 2 | Explain the scientific background and rationale for the investigation being reported | 3 | However, the large participants samples in a phase 3 trial.. |
| Objectives | 3 | State specific objectives, including any prespecified hypotheses | 3 | Following ANVISA recommendations, IB undertook a post-market approval safety evaluation to characterize AEFI…. |
| Methods | | | |  |
| Study design | 4 | Present key elements of study design early in the paper | 3 | This phase IV prospective observational study …. |
| Setting | 5 | Describe the setting, locations, and relevant dates, including periods of recruitment, exposure, follow-up, and data collection | 4 | Ethical approval was obtained from the coordinating study site (University of São Paulo, …) |
| Participants | 6 | (*a*) *Cohort study*—Give the eligibility criteria, and the sources and methods of selection of participants. Describe methods of follow-up  *Case-control study*—Give the eligibility criteria, and the sources and methods of case ascertainment and control selection. Give the rationale for the choice of cases and controls  *Cross-sectional study*—Give the eligibility criteria, and the sources and methods of selection of participants | 4 | Eligible participants for the CoronaVac vaccine, aged 18-59 years-old… |
|  |  | (*b*) *Cohort study*—For matched studies, give matching criteria and number of exposed and unexposed  *Case-control study*—For matched studies, give matching criteria and the number of controls per case |  |  |
| Variables | 7 | Clearly define all outcomes, exposures, predictors, potential confounders, and effect modifiers. Give diagnostic criteria, if applicable | 6 | In this context, an adverse event was defined as any undesirable medical occurrence that affected a vaccinated participant, …. |
| Data sources/ measurement | 8* | For each variable of interest, give sources of data and details of methods of assessment (measurement). Describe comparability of assessment methods if there is more than one group | *7* | *The severity of an AE was classified from grade one to four according to the Toxicity Grading Scale for Healthy Adult and Adolescent Volunteers enrolled ….* |
| Bias | 9 | Describe any efforts to address potential sources of bias | 7 | The study staff was instructed to incorporate the following information in their AE evaluations: the classification… |
| Study size | 10 | Explain how the study size was arrived at | 8 | The sample size was estimated following the recommendation of the Guide to Clinical Evaluations of New Vaccines… |

Continued on next page

| Quantitative variables | 11 | Explain how quantitative variables were handled in the analyses. If applicable, describe which groupings were chosen and why | 8 | Descriptive statistical data analysis of AE and AR was performed, stratified by study group (younger and older adults)… |
| --- | --- | --- | --- | --- |
| Statistical methods | 12 | (*a*) Describe all statistical methods, including those used to control for confounding | 8 | Descriptive statistical data analysis of AE and AR was performed, stratified by study group (younger and older adults)… |
|  |  | (*b*) Describe any methods used to examine subgroups and interactions | N/A |  |
|  |  | (*c*) Explain how missing data were addressed | 8 | Missing data were handled with… |
|  |  | (*d*) *Cohort study*—If applicable, explain how loss to follow-up was addressed  *Case-control study*—If applicable, explain how matching of cases and controls was addressed  *Cross-sectional study*—If applicable, describe analytical methods taking account of sampling strategy | N/A |  |
|  |  | (*e*) Describe any sensitivity analyses | N/A |  |
| Results | | | | |
| Participants | 13* | (a) Report numbers of individuals at each stage of study—e.g. numbers potentially eligible, examined for eligibility, confirmed eligible, included in the study, completing follow-up, and analysed | 9 | Figure 1 presents the study participants´ flowchart, …. |
|  |  | (b) Give reasons for non-participation at each stage | 9 | Figure 1 presents the study participants´ flowchart, …. |
|  |  | (c) Consider use of a flow diagram | 9 | Figure 1 presents the study participants´ flowchart, …. |
| Descriptive data | 14* | (a) Give characteristics of study participants (e.g. demographic, clinical, social) and information on exposures and potential confounders | 9 | The study incorporated a total of 538 participants, where young adults …. |
|  |  | (b) Indicate number of participants with missing data for each variable of interest | N/A |  |
|  |  | (c) *Cohort study*—Summarise follow-up time (e.g., average and total amount) | N/A |  |
| Outcome data | 15* | *Cohort study*—Report numbers of outcome events or summary measures over time |  |  |
|  |  | *Case-control study—*Report numbers in each exposure category, or summary measures of exposure |  |  |
|  |  | *Cross-sectional study—*Report numbers of outcome events or summary measures | *19* | *Table 2. Frequency of adverse events and adverse reactions after CoronaVac vaccination* |
| Main results | 16 | (*a*) Give unadjusted estimates and, if applicable, confounder-adjusted estimates and their precision (e.g., 95% confidence interval). Make clear which confounders were adjusted for and why they were included | N/A |  |
|  |  | (*b*) Report category boundaries when continuous variables were categorized | N/A |  |
|  |  | (*c*) If relevant, consider translating estimates of relative risk into absolute risk for a meaningful time period | N/A |  |

Continued on next page

| Other analyses | 17 | Report other analyses done—e.g. analyses of subgroups and interactions, and sensitivity analyses | 11 | Exploratory endpoints |
| --- | --- | --- | --- | --- |
| Discussion | | | | |
| Key results | 18 | Summarise key results with reference to study objectives | 11 | Our findings suggest that most AE and AR following vaccination were mild to moderate in severity and resolved spontaneously. |
| Limitations | 19 | Discuss limitations of the study, taking into account sources of potential bias or imprecision. Discuss both direction and magnitude of any potential bias | 12 | Our study has limitations. Sample size did not reach the previously calculated number…. |
| Interpretation | 20 | Give a cautious overall interpretation of results considering objectives, limitations, multiplicity of analyses, results from similar studies, and other relevant evidence | 13 | Our study adds to the growing body of evidence on the safety of … |
| Generalisability | 21 | Discuss the generalisability (external validity) of the study results | 13 | In conclusion, our data substantiate the overall safety of the CoronaVac. |
| Other information | |  | | |
| Funding | 22 | Give the source of funding and the role of the funders for the present study and, if applicable, for the original study on which the present article is based | 14 | This study was sponsored by Fundação Butantan |

*Give information separately for cases and controls in case-control studies and, if applicable, for exposed and unexposed groups in cohort and cross-sectional studies.

**Note:** An Explanation and Elaboration article discusses each checklist item and gives methodological background and published examples of transparent reporting. The STROBE checklist is best used in conjunction with this article (freely available on the Web sites of PLoS GPH at https://journals.plos.org/globalpublichealth/s/submission-guidelines#loc-guidelines-for-specific-study-types/, Annals of Internal Medicine at http://www.annals.org/, and Epidemiology at http://www.epidem.com/). Information on the STROBE Initiative is available at www.strobe-statement.org.
