# Supplementary material for: Evaluating the safety profile of the CoronaVac in adult and older adult populations: A phase IV prospective observational study in Brazil
Source: PLOS Glob Public Health. 2025 Feb 25;5(2):e0004069. doi: 10.1371/journal.pgph.0004069 (PMC12048030; doi:10.1371/journal.pgph.0004069)
Supplement: S1 Appendix — (DOCX) [file pgph.0004069.s002.docx]

**Supplementary Appendix**

**Evaluating the safety profile of the CoronaVac in adult and older adult populations: A Phase IV prospective observational study in Brazil**

**Authors**: Vanessa Infante^1^, Monica Akissue de Camargo Teixeira Cintra^1^, Eder Gatti Fernandes^1^, Ana Paula Loch^1^, Lucas Ragiotto^1^, Patrícia Emília Braga^1^, Maria da Graça Salomão^1^, Maria Beatriz Bastos Lucchesi^1^, Mayra Martho Moura de Oliveira^1^, Vera Lúcia Gattás^1^, Anderson Soares da Silva^2^, Paulo José Fortes Villas Boas^3^, Marta Heloisa Lopes^4^, José Moreira^1*^, Fernanda Castro Boulos^1^, CFV-01-IB study group^#^

**Authors’ affiliations**

^1^ Clinical Trials and Pharmacovigilance Center, Instituto Butantan, São Paulo, Brazil.

^2^ Centro de Saúde Escola da Faculdade de Medicina de Ribeirão Preto da Universidade de São Paulo (HCFMRP-USP) Dr. Joel Domingos Machado.

^3^ Centro de Saúde Escola da Faculdade de Medicina de Botucatu – Unesp.

^4^ Centro de Referência de Imunobiológicos Especiais Hospital das Clínicas da Faculdade de Medicina da Universidade de São Paulo (CRIE-HCFMUSP).

^#^ Members of the CFV-01-IB study group is provided in the Acknowledgement’.

**Corresponding author**:

* jose.amoreira@fundacaobutantan.org.br

**TABLE OF CONTENTS**

S1. Checklist - STROBE- v4-combined-PlosGPG_CFV_adults

S1. Appendix - Severity classification 4

# S2. Appendix Causal Relationship Classification 7

S1. Table - Frequency and severity of Adverse Reactions, solicited (local and systemic) and unsolicited, occurring up to 7 days after administration of each vaccine dose in adults (18 to 59 years), according to severity 8

S2. Table - Frequency and severity of Adverse Reactions, solicited (local and systemic) and unsolicited, occurring up to 7 days after administration of each vaccine dose in older adults (≥60 years), according to severity 12

S3. Table - Frequency of Adverse Reactions, solicited (local and systemic) and unsolicited, occurring within 30 minutes after administration of each vaccine dose in a.) adults (18 to 59 years) and b.) older adults (≥60 years), according to severity 14

A. ADULTS (18 TO 59 YEARS) 14

B. OLDER ADULTS (≥60 YEARS) 15

S4. Table - Frequency of solicited (local and systemic) and unsolicited adverse reactions which required medical attention, occuring at any time within 42 days after administration of each vaccine dose in a.) adults (18 to 59 years) and b.) older adults (≥ 60 years), according to severity 17

A. ADULTS (18 TO 59 YEARS) 17

B. OLDER ADULTS (≥60 YEARS) 18

S5. Table 5 Serious Adverse Events (SAE) and Adverse Events of Special Interest (AESI) according to the administration dose regarding description (MedDRA code), causality, severity, predctability and outcomes 20

S6. Table - Adverse Event (AE) details observed in pregnant women and newborn according to the administrantion dose regarding description (MedDRA code), causality, severity, predictability and outcomes 21

References 22

# S1. Appendix Severity classification

Based on the guide “Toxicity Grading Scale for Healthy Adult and Adolescent Volunteers Enrolled in Preventive Vaccine Clinical Trials” from the US Food and Drug Administration (USFDA).^1, 2, 3^

Classification of the severity of the solicited clinical adverse events.

| **Adverse Event** | **Grade 1** | **Grade 2** | **Grade 3** | **Grade 4** |
| --- | --- | --- | --- | --- |
| Pain at the injection site of the investigational product | Does not interfere  with daily activities | Repeated use of non-narcotic analgesic >24 hours  OR  Mild interference with daily activities | Any use of a narcotic analgesic  OR  Prevent daily activities | Emergency department visit*  OR  Hospitalization |
| Erythema at the injection site of the investigational product ^†^ | 25 – 50 mm | 51 – 100 mm | >100 mm | Necrosis  OR  Exfoliative dermatitis |
| Swelling at the injection site of the investigational product ^†^ | 25 – 50 mm | 51 – 100 mm  OR  Mild interference with daily activities | >100 mm | Necrosis |
| Induration at the injection site of the investigational product ^†^ | 25 – 50 mm | 51 – 100 mm  OR  Mild interference with daily activities | >100 mm | Necrosis |
| Pruritus at the injection site of the investigational product | Does not interfere  with daily activities | Mild interference with daily activities | Prevent daily activities | Emergency department visit*  OR  Hospitalization |
| Nausea | Does not interfere  with daily activities  OR  1 to 2 episodes in 24 hours | Mild interference with daily activities  OR  More than 2 episodes in 24 hours | Prevent daily activities. it requires IV hydration | Emergency department visit*  OR  Hospitalization  OR  Hypovolemic shock |
| Vomiting | Does not interfere  with daily activities  OR  1 to 2 episodes in 24 hours | Mild interference with daily activities  OR  More than 2 episodes in 24 hours | Prevent daily activities. it requires IV hydration | Emergency department visit*  OR  Hospitalization  OR  Hypovolemic shock |
| Fever | 37.8 – 38.4°C | 38.5 – 38.9°C | 39.0 – 40.0°C | >40°C |
| Chills | Slight cold sensation; chills. teeth chatter | Moderate chills in the entire body. it requires the use of narcotics | Serious or prolonged. no response to narcotics | ----- |
| Headache | Does not interfere  with daily activities | Repeated use of non-narcotic analgesic >24 hours  OR  Mild interference with daily activities | Any use of a narcotic analgesic  OR  Prevent daily activities | Emergency department visit*  OR  Hospitalization |
| Diarrhea | Increase of <4 stools per day  over baseline; mild increase in  ostomy output compared to  baseline | Increase of 4 - 6 stools per  day over baseline; moderate  increase in ostomy output  compared to baseline;  limiting instrumental ADL | Increase of >=7 stools per day  over baseline; hospitalization  indicated; severe increase in  ostomy output compared to  baseline; limiting self care  ADL | Life-threatening  consequences; urgent  intervention indicated |
| Anorexia | Loss of appetite without changing in eating habits | Oral intake altered without  significant weight loss or  malnutrition; oral nutritional  supplements indicated | Associated with significant  weight loss or malnutrition  (e.g., inadequate oral caloric  and/or fluid intake); tube  feeding or TPN indicated | Life-threatening  consequences; urgent  intervention indicated |
| Fatigue | Does not interfere  with daily activities | Mild interference with daily activities | Prevent daily activities | Emergency department visit*  OR  Hospitalization |
| Myalgia | Does not interfere  with daily activities | Mild interference with daily activities | Prevent daily activities | Emergency department visit*  OR  Hospitalization |
| Arthralgia | Does not interfere  with daily activities | Mild interference with daily activities | Prevent daily activities | Emergency department visit*  OR  Hospitalization |
| Rash maculo-papular^‡^ | Macules/papules covering  <10% BSA with or without  symptoms (e.g., pruritus,  burning, tightness) | Macules/papules covering 10  - 30% BSA with or without  symptoms (e.g., pruritus,  burning, tightness); limiting  instrumental ADL; rash  covering > 30% BSA with or  without mild symptoms | Macules/papules covering  >30% BSA with moderate or  severe symptoms; limiting self  care ADL | Emergency department visit*  OR  Hospitalization |
| Pruritus | Mild or localized; topical  intervention indicated | Widespread and intermittent;  skin changes from scratching  (e.g., edema, papulation,  excoriations, lichenification,  oozing/crusts); oral  intervention indicated;  limiting instrumental ADL | Widespread and constant;  limiting self care ADL or sleep;  systemic corticosteroid or  immunosuppressive therapy  indicated | Emergency department visit*  OR  Hospitalization |
| Cough | Mild symptoms;  nonprescription intervention  indicated | Moderate symptoms, medical  intervention indicated;  limiting instrumental ADL | Severe symptoms; limiting  self care ADL | Emergency department visit*  OR  Hospitalization |
| Allergic reaction | Systemic intervention not  indicated | Oral intervention indicated | Bronchospasm;  hospitalization indicated for  clinical sequelae; intravenous  intervention indicated | Life-threatening  consequences; urgent  intervention indicated |

* It requires 12 hours or more of admission to a ward or emergency department for the management of the adverse event.

^†^ The value recorded should be measured at the largest diameter and as a continuous variable.

^‡^ Specify whether the skin rash is located in any region of the body or if it is widespread.

# References

[1] CBER/USFDA/USDHHS Guidance for Industry: Toxicity Grading Scale for Healthy Adult and Adolescent Volunteers Enrolled in Preventive Vaccine Clinical Trials [Internet]. Silver Spring: US Food and Drug Administration; 2007 [cited 2011 Set 29]. Available at: http://www.fda.gov/downloads/BiologicsBloodVaccines/GuidanceComplianceRegulatoryInformation/Guidances/Vaccines/ucm091977.pdf.

[2] ICH Clinical Safety Data management: Definitions and Standards for Expedited Reporting [Internet]. Genebra: International Conference on Harmonisation of Technical Requirements ror Registration of Pharmaceuticals for Human Use; 1994 [cited 2012 Oct 15]. E2A. Available at: http://www.ich.org/fileadmin/Public_Web_Site/ICH_Products/Guidelines/Efficacy/E2A/Step4/E2A_Guideline.pdf.

[3] NIC/NIH NCI Common Terminology Criteria for Adverse Events (CTCAE) [Internet]. Available at: http://evs.nci.nih.gov/ftp1/CTCAE/About.html.

[4] UMC/WHO The use of the WHO-UMC system for standardised case causality assessment [Internet]. Uppsala:The Uppsala Monitoring Centre. Available at: http://www.who-umc.org/Graphics/24734.pdf.
