# Supplementary material for: Evaluating the safety profile of the CoronaVac in adult and older adult populations: A phase IV prospective observational study in Brazil
Source: PLOS Glob Public Health. 2025 Feb 25;5(2):e0004069. doi: 10.1371/journal.pgph.0004069 (PMC12048030; doi:10.1371/journal.pgph.0004069)
Supplement: S3 Table — (DOCX) [file pgph.0004069.s006.docx]

**Supplementary Appendix**

**Evaluating the safety profile of the CoronaVac in adult and older adult populations: A Phase IV prospective observational study in Brazil**

**Authors**: Vanessa Infante^1^, Monica Akissue de Camargo Teixeira Cintra^1^, Eder Gatti Fernandes^1^, Ana Paula Loch^1^, Lucas Ragiotto^1^, Patrícia Emília Braga^1^, Maria da Graça Salomão^1^, Maria Beatriz Bastos Lucchesi^1^, Mayra Martho Moura de Oliveira^1^, Vera Lúcia Gattás^1^, Anderson Soares da Silva^2^, Paulo José Fortes Villas Boas^3^, Marta Heloisa Lopes^4^, José Moreira^1*^, Fernanda Castro Boulos^1^, CFV-01-IB study group^#^

**Authors’ affiliations**

^1^ Clinical Trials and Pharmacovigilance Center, Instituto Butantan, São Paulo, Brazil.

^2^ Centro de Saúde Escola da Faculdade de Medicina de Ribeirão Preto da Universidade de São Paulo (HCFMRP-USP) Dr. Joel Domingos Machado.

^3^ Centro de Saúde Escola da Faculdade de Medicina de Botucatu – Unesp.

^4^ Centro de Referência de Imunobiológicos Especiais Hospital das Clínicas da Faculdade de Medicina da Universidade de São Paulo (CRIE-HCFMUSP).

^#^ Members of the CFV-01-IB study group is provided in the Acknowledgement’.

**Corresponding author**:

* jose.amoreira@fundacaobutantan.org.br

**TABLE OF CONTENTS**

S1. Checklist-- STROBE- v4-combined-PlosGPG_CFV_adults

S1. Appendix - Severity classification 4

# S2. Appendix Causal Relationship Classification 7

S1. Table - Frequency and severity of Adverse Reactions, solicited (local and systemic) and unsolicited, occurring up to 7 days after administration of each vaccine dose in adults (18 to 59 years), according to severity 8

S2. Table - Frequency and severity of Adverse Reactions, solicited (local and systemic) and unsolicited, occurring up to 7 days after administration of each vaccine dose in older adults (≥60 years), according to severity 12

S3. Table - Frequency of Adverse Reactions, solicited (local and systemic) and unsolicited, occurring within 30 minutes after administration of each vaccine dose in a.) adults (18 to 59 years) and b.) older adults (≥60 years), according to severity 14

A. ADULTS (18 TO 59 YEARS) 14

B. OLDER ADULTS (≥60 YEARS) 15

S4. Table - Frequency of solicited (local and systemic) and unsolicited adverse reactions which required medical attention, occuring at any time within 42 days after administration of each vaccine dose in a.) adults (18 to 59 years) and b.) older adults (≥ 60 years), according to severity 17

A. ADULTS (18 TO 59 YEARS) 17

B. OLDER ADULTS (≥60 YEARS) 18

S5. Table 5 Serious Adverse Events (SAE) and Adverse Events of Special Interest (AESI) according to the administration dose regarding description (MedDRA code), causality, severity, predctability and outcomes 20

S6. Table - Adverse Event (AE) details observed in pregnant women and newborn according to the administrantion dose regarding description (MedDRA code), causality, severity, predictability and outcomes 21

References 22

S3. table Frequency of Adverse Reactions, solicited (local and systemic) and unsolicited, occurring within 30 minutes after administration of each vaccine dose in a.) adults (18 to 59 years) and b.) older adults (≥60 years), according to severity

## ADULTS (18 TO 59 YEARS)

| **Adverse Reaction** | $\boldsymbol{1}^{\boldsymbol{st}}$ **DOSE** | | | | | | | | | | | | | $\boldsymbol{2}^{\boldsymbol{nd}}$**DOSE** | | | | | | | | | | | | |
| --- | --- | --- | --- | --- | --- | --- | --- | --- | --- | --- | --- | --- | --- | --- | --- | --- | --- | --- | --- | --- | --- | --- | --- | --- | --- | --- |
|  | **Adults**  **with AR (n=487)** | | **Total**  **AR** | **Grade**  **0** | | **Grade**  **1** | | **Grade**  **2** | | **Grade**  **3** | | **Grade**  **4** | | **Adults with AR (n=438)** | | **Total**  **AR** | **Grade**  **0** | | **Grade**  **1** | | **Grade**  **2** | | **Grade**  **3** | | **Grade**  **4** | |
|  | **n** | **%** | **n** | **n** | **%** | **n** | **%** | **n** | **%** | **n** | **%** | **n** | **%** | **n** | **%** | **n** | **n** | **%** | **n** | **%** | **n** | **%** | **n** | **%** | **n** | **%** |
| **SOLICITED** |  |  |  |  |  |  |  |  |  |  |  |  |  |  |  |  |  |  |  |  |  |  |  |  |  |  |
| **Local** |  |  |  |  |  |  |  |  |  |  |  |  |  |  |  |  |  |  |  |  |  |  |  |  |  |  |
| Pain | 85 | 17.5 | 85 | 0 | 0.0 | 78 | 91.8 | 5 | 5.9 | 2 | 2.4 | 0 | 0.0 | 52 | 11.9 | 52 | 0 | 0.0 | 51 | 98.1 | 1 | 1.9 | 0 | 0.0 | 0 | 0.0 |
| Erythema | 2 | 0.4 | 2 | 0 | 0.0 | 2 | 100 | 0 | 0.0 | 0 | 0.0 | 0 | 0.0 | 2 | 0.5 | 2 | 0 | 0.0 | 2 | 100 | 0 | 0.0 | 0 | 0.0 | 0 | 0.0 |
| Induration | 5 | 1.0 | 5 | 1 | 20.0 | 3 | 60.0 | 1 | 20.0 | 0 | 0.0 | 0 | 0.0 | 3 | 0.7 | 3 | 1 | 33.3 | 2 | 66.7 | 0 | 0.0 | 0 | 0.0 | 0 | 0.0 |
| Pruritus | 1 | 0.2 | 1 | 0 | 0.0 | 1 | 100 | 0 | 0.0 | 0 | 0.0 | 0 | 0.0 | 1 | 0.2 | 1 | 0 | 0.0 | 1 | 100 | 0 | 0.0 | 0 | 0.0 | 0 | 0.0 |
| Tenderness | 48 | 9.9 | 48 | 0 | 0.0 | 45 | 93.8 | 2 | 4.2 | 1 | 2.1 | 0 | 0.0 | 32 | 7.3 | 32 | 0 | 0.0 | 31 | 96.9 | 1 | 3.1 | 0 | 0.0 | 0 | 0.0 |
| **Systemic** |  |  |  |  |  |  |  |  |  |  |  |  |  |  |  |  |  |  |  |  |  |  |  |  |  |  |
| Arthralgia | 0 | 0.0 | 0 | 0 | 0.0 | 0 | 0.0 | 0 | 0.0 | 0 | 0.0 | 0 | 0.0 | 1 | 0.2 | 1 | 0 | 0.0 | 1 | 100 | 0 | 0.0 | 0 | 0.0 | 0 | 0.0 |
| Headache | 9 | 1.8 | 9 | 0 | 0.0 | 6 | 66.7 | 2 | 22.2 | 1 | 11.1 | 0 | 0.0 | 4 | 0.9 | 4 | 0 | 0.0 | 3 | 75.0 | 1 | 25.0 | 0 | 0.0 | 0 | 0.0 |
| Fatigue | 3 | 0.6 | 3 | 0 | 0.0 | 1 | 33.3 | 0 | 0.0 | 2 | 66.7 | 0 | 0.0 | 3 | 0.7 | 3 | 0 | 0.0 | 3 | 100 | 0 | 0.0 | 0 | 0.0 | 0 | 0.0 |
| Myalgia | 3 | 0.6 | 3 | 0 | 0.0 | 1 | 33.3 | 1 | 33.3 | 1 | 33.3 | 0 | 0.0 | 1 | 0.2 | 1 | 0 | 0.0 | 1 | 100 | 0 | 0.0 | 0 | 0.0 | 0 | 0.0 |
| Nausea | 3 | 0.6 | 3 | 0 | 0.0 | 3 | 100 | 0 | 0.0 | 0 | 0.0 | 0 | 0.0 | 2 | 0.5 | 2 | 0 | 0.0 | 1 | 50.0 | 1 | 50.0 | 0 | 0.0 | 0 | 0.0 |
| Cough | 1 | 0.2 | 1 | 0 | 0.0 | 1 | 100 | 0 | 0.0 | 0 | 0.0 | 0 | 0.0 | 1 | 0.2 | 1 | 0 | 0.0 | 1 | 100 | 0 | 0.0 | 0 | 0.0 | 0 | 0.0 |
| Vomiting | 0 | 0.0 | 0 | 0 | 0.0 | 0 | 0.0 | 0 | 0.0 | 0 | 0.0 | 0 | 0.0 | 0 | 0.0 | 0 | 0 | 0.0 | 0 | 0.0 | 0 | 0.0 | 0 | 0.0 | 0 | 0.0 |
| **TOTAL SOLICITED** | 129 | 26.5 | 160 | 1 | 0.6 | 141 | 88.1 | 11 | 6.9 | 7 | 4.4 | 0 | 0.0 | 84 | 19.2 | 102 | 1 | 1.0 | 97 | 95.1 | 4 | 3.9 | 0 | 0.0 | 0 | 0.0 |
| **UNSOLICITED** |  |  |  |  |  |  |  |  |  |  |  |  |  |  |  |  |  |  |  |  |  |  |  |  |  |  |
| Dry mouth | 0 | 0.0 | 0 | 0 | 0.0 | 0 | 0.0 | 0 | 0.0 | 0 | 0.0 | 0 | 0.0 | 1 | 0.2 | 1 | 0 | 0.0 | 1 | 100 | 0 | 0.0 | 0 | 0.0 | 0 | 0.0 |
| Heat at the administration site | 0 | 0.0 | 0 | 0 | 0.0 | 0 | 0.0 | 0 | 0.0 | 0 | 0.0 | 0 | 0.0 | 1 | 0.2 | 1 | 0 | 0.0 | 1 | 100 | 0 | 0.0 | 0 | 0.0 | 0 | 0.0 |
| Oropharyngeal discomfort | 1 | 0.2 | 1 | 0 | 0.0 | 1 | 100 | 0 | 0.0 | 0 | 0.0 | 0 | 0.0 | 1 | 0.2 | 1 | 0 | 0.0 | 1 | 100 | 0 | 0.0 | 0 | 0.0 | 0 | 0.0 |
| Axillary pain | 1 | 0.2 | 1 | 0 | 0.0 | 1 | 100 | 0 | 0.0 | 0 | 0.0 | 0 | 0.0 | 0 | 0.0 | 0 | 0 | 0.0 | 0 | 0.0 | 0 | 0.0 | 0 | 0.0 | 0 | 0.0 |
| Ecchymosis at the administration site | 0 | 0.0 | 0 | 0 | 0.0 | 0 | 0.0 | 0 | 0.0 | 0 | 0.0 | 0 | 0.0 | 1 | 0.2 | 1 | 0 | 0.0 | 1 | 100 | 0 | 0.0 | 0 | 0.0 | 0 | 0.0 |
| Stove | 0 | 0.0 | 0 | 0 | 0.0 | 0 | 0.0 | 0 | 0.0 | 0 | 0.0 | 0 | 0.0 | 0 | 0.0 | 0 | 0 | 0.0 | 0 | 0 | 0 | 0.0 | 0 | 0.0 | 0 | 0.0 |
| Paresthesia at the administration site | 3 | 0.6 | 3 | 0 | 0.0 | 3 | 100 | 0 | 0.0 | 0 | 0.0 | 0 | 0.0 | 1 | 0.2 | 1 | 0 | 0.0 | 1 | 100 | 0 | 0.0 | 0 | 0.0 | 0 | 0.0 |
| Gastroesophageal reflux | 0 | 0.0 | 0 | 0 | 0.0 | 0 | 0.0 | 0 | 0.0 | 0 | 0.0 | 0 | 0.0 | 0 | 0.0 | 0 | 0 | 0.0 | 0 | 0.0 | 0 | 0.0 | 0 | 0.0 | 0 | 0.0 |
| Somnolence | 2 | 0.4 | 2 | 0 | 0.0 | 2 | 100 | 0 | 0.0 | 0 | 0.0 | 0 | 0.0 | 0 | 0.0 | 0 | 0 | 0.0 | 0 | 0.0 | 0 | 0.0 | 0 | 0.0 | 0 | 0.0 |
| Dizziness | 3 | 0.6 | 3 | 0 | 0.0 | 3 | 100 | 0 | 0.0 | 0 | 0.0 | 0 | 0.0 | 1 | 0.2 | 1 | 0 | 0.0 | 1 | 100 | 0 | 0.0 | 0 | 0.0 | 0 | 0.0 |
| Vertigo | 0 | 0.0 | 0 | 0 | 0.0 | 0 | 0.0 | 0 | 0.0 | 0 | 0.0 | 0 | 0.0 | 1 | 0.2 | 1 | 0 | 0.0 | 1 | 100 | 0 | 0.0 | 0 | 0.0 | 0 | 0.0 |
| **TOTAL UNSOLICITED** | 9 | 1.8 | 10 | 0 | 0.0 | 10 | 100 | 0 | 0.0 | 0 | 0.0 | 0 | 0.0 | 7 | 1.6 | 7 | 0 | 0.0 | 7 | 100 | 0 | 0.0 | 0 | 0.0 | 0 | 0.0 |
| AR: Adverse Reaction | | | | | | | | | | | | | | | | | | | | | | | | | | |

## OLDER ADULTS (≥60 YEARS)

| **Adverse Reaction** | $\boldsymbol{1}^{\boldsymbol{st}}$ **DOSE** | | | | | | | | | | | $\boldsymbol{2}^{\boldsymbol{nd}}$**DOSE** | | | | | | | | | | |
| --- | --- | --- | --- | --- | --- | --- | --- | --- | --- | --- | --- | --- | --- | --- | --- | --- | --- | --- | --- | --- | --- | --- |
|  | **Older adults with AR (n=51)** | | **Total**  **AR** | **Grade**  **1** | | **Grade**  **2** | | **Grade**  **3** | | **Grade**  **4** | | **Older adults with AR (n=50)** | | **Total**  **AR** | **Grade**  **1** | | **Grade**  **2** | | **Grade**  **3** | | **Grade**  **4** | |
|  | **n** | **%** | **n** | **n** | **%** | **n** | **%** | **n** | **%** | **n** | **%** | **n** | **%** | **n** | **n** | **%** | **n** | **%** | **n** | **%** | **n** | **%** |
| **SOLICITED** |  |  |  |  |  |  |  |  |  |  |  |  |  |  |  |  |  |  |  |  |  |  |
| **Local** |  |  |  |  |  |  |  |  |  |  |  |  |  |  |  |  |  |  |  |  |  |  |
| Pain | 4 | 7.8 | 4 | 4 | 100 | 0 | 0.0 | 0 | 0.0 | 0 | 0.0 | 6 | 12.0 | 6 | 6 | 100 | 0 | 0.0 | 0 | 0.0 | 0 | 0.0 |
| Erythema | 0 | 0.0 | 0 | 0 | 0 | 0 | 0.0 | 0 | 0.0 | 0 | 0.0 | 0 | 0.0 | 0 | 0 | 0 | 0 | 0.0 | 0 | 0.0 | 0 | 0.0 |
| Induration | 0 | 0.0 | 0 | 0 | 0 | 0 | 0.0 | 0 | 0.0 | 0 | 0.0 | 0 | 0.0 | 0 | 0 | 0 | 0 | 0.0 | 0 | 0.0 | 0 | 0.0 |
| Pruritus | 0 | 0.0 | 0 | 0 | 0 | 0 | 0.0 | 0 | 0.0 | 0 | 0.0 | 1 | 2.0 | 1 | 0 | 0 | 1 | 100 | 0 | 0.0 | 0 | 0.0 |
| Tenderness | 2 | 3.9 | 2 | 2 | 100 | 0 | 0.0 | 0 | 0.0 | 0 | 0.0 | 1 | 2.0 | 1 | 1 | 100 | 0 | 0.0 | 0 | 0.0 | 0 | 0.0 |
| **Systemic** |  |  |  |  |  |  |  |  |  |  |  |  |  |  |  |  |  |  |  |  |  |  |
| Arthralgia | 0 | 0.0 | 0 | 0 | 0.0 | 0 | 0.0 | 0 | 0.0 | 0 | 0.0 | 1 | 2.0 | 1 | 1 | 100 | 0 | 0.0 | 0 | 0.0 | 0 | 0.0 |
| Headache | 1 | 2.0 | 1 | 1 | 100 | 0 | 0.0 | 0 | 0.0 | 0 | 0.0 | 0 | 0.0 | 0 | 0 | 0.0 | 0 | 0.0 | 0 | 0.0 | 0 | 0.0 |
| Fatigue | 0 | 0.0 | 0 | 0 | 0.0 | 0 | 0.0 | 0 | 0.0 | 0 | 0.0 | 0 | 0.0 | 0 | 0 | 0 | 0 | 0.0 | 0 | 0.0 | 0 | 0.0 |
| Myalgia | 0 | 0.0 | 0 | 0 | 0.0 | 0 | 0.0 | 0 | 0.0 | 0 | 0.0 | 0 | 0.0 | 0 | 0 | 0 | 0 | 0.0 | 0 | 0.0 | 0 | 0.0 |
| Nausea | 0 | 0.0 | 0 | 0 | 0 | 0 | 0.0 | 0 | 0.0 | 0 | 0.0 | 0 | 0.0 | 0 | 0 | 0.0 | 0 | 0.0 | 0 | 0.0 | 0 | 0.0 |
| Cough | 0 | 0.0 | 0 | 0 | 0 | 0 | 0.0 | 0 | 0.0 | 0 | 0.0 | 0 | 0.0 | 0 | 0 | 0 | 0 | 0.0 | 0 | 0.0 | 0 | 0.0 |
| Vomiting | 0 | 0.0 | 0 | 0 | 0.0 | 0 | 0.0 | 0 | 0.0 | 0 | 0.0 | 0 | 0.0 | 0 | 0 | 0.0 | 0 | 0.0 | 0 | 0.0 | 0 | 0.0 |
| **TOTAL SOLICITED** | 6 | 11.8 | 7 | 7 | 100 | 0 | 0.0 | 0 | 0.0 | 0 | 0.0 | 6 | 12.0 | 9 | 8 | 88.9 | 1 | 11.1 | 0 | 0.0 | 0 | 0.0 |
| **UNSOLICITED** |  |  |  |  |  |  |  |  |  |  |  |  |  |  |  |  |  |  |  |  |  |  |
| Dry mouth | 1 | 2.0 | 1 | 1 | 100 | 0 | 0.0 | 0 | 0.0 | 0 | 0.0 | 0 | 0.0 | 0 | 0 | 0.0 | 0 | 0.0 | 0 | 0.0 | 0 | 0.0 |
| Heat at the administration site | 0 | 0.0 | 0 | 0 | 0.0 | 0 | 0.0 | 0 | 0.0 | 0 | 0.0 | 0 | 0.0 | 0 | 0 | 0.0 | 0 | 0.0 | 0 | 0.0 | 0 | 0.0 |
| Oropharyngeal discomfort | 0 | 0.0 | 0 | 0 | 0.0 | 0 | 0.0 | 0 | 0.0 | 0 | 0.0 | 0 | 0.0 | 0 | 0 | 0.0 | 0 | 0.0 | 0 | 0.0 | 0 | 0.0 |
| Axillary pain | 0 | 0.0 | 0 | 0 | 0.0 | 0 | 0.0 | 0 | 0.0 | 0 | 0.0 | 0 | 0.0 | 0 | 0 | 0.0 | 0 | 0.0 | 0 | 0.0 | 0 | 0.0 |
| Ecchymosis at the administration site | 0 | 0.0 | 0 | 0 | 0.0 | 0 | 0.0 | 0 | 0.0 | 0 | 0.0 | 0 | 0.0 | 0 | 0 | 0.0 | 0 | 0.0 | 0 | 0.0 | 0 | 0.0 |
| Stove | 1 | 2.0 | 1 | 1 | 100 | 0 | 0.0 | 0 | 0.0 | 0 | 0.0 | 0 | 0.0 | 0 | 0 | 0 | 0 | 0.0 | 0 | 0.0 | 0 | 0.0 |
| Paresthesia at the administration site | 0 | 0.0 | 0 | 0 | 0.0 | 0 | 0.0 | 0 | 0.0 | 0 | 0.0 | 0 | 0.0 | 0 | 0 | 0.0 | 0 | 0.0 | 0 | 0.0 | 0 | 0.0 |
| Gastroesophageal reflux | 1 | 2.0 | 1 | 0 | 0 | 1 | 100 | 0 | 0.0 | 0 | 0.0 | 0 | 0.0 | 0 | 0 | 0 | 0 | 0.0 | 0 | 0.0 | 0 | 0.0 |
| Somnolence | 0 | 0.0 | 0 | 0 | 0.0 | 0 | 0.0 | 0 | 0.0 | 0 | 0.0 | 0 | 0.0 | 0 | 0 | 0.0 | 0 | 0.0 | 0 | 0.0 | 0 | 0.0 |
| Dizziness | 0 | 0.0 | 0 | 0 | 0.0 | 0 | 0.0 | 0 | 0.0 | 0 | 0.0 | 0 | 0.0 | 0 | 0 | 0.0 | 0 | 0.0 | 0 | 0.0 | 0 | 0.0 |
| Vertigo | 0 | 0.0 | 0 | 0 | 0.0 | 0 | 0.0 | 0 | 0.0 | 0 | 0.0 | 0 | 0.0 | 0 | 0 | 0.0 | 0 | 0.0 | 0 | 0.0 | 0 | 0.0 |
| **TOTAL UNSOLICITED** | 1 | 2.0 | 3 | 2 | 67 | 1 | 33.3 | 0 | 0.0 | 0 | 0.0 | 0 | 0.0 | 0 | 0 | 0 | 0 | 0.0 | 0 | 0.0 | 0 | 0.0 |
| AR: Adverse Reaction | | | | | | | | | | | | | | | | | | | | | | |

# References

[1] CBER/USFDA/USDHHS Guidance for Industry: Toxicity Grading Scale for Healthy Adult and Adolescent Volunteers Enrolled in Preventive Vaccine Clinical Trials [Internet]. Silver Spring: US Food and Drug Administration; 2007 [cited 2011 Set 29]. Available at: http://www.fda.gov/downloads/BiologicsBloodVaccines/GuidanceComplianceRegulatoryInformation/Guidances/Vaccines/ucm091977.pdf.

[2] ICH Clinical Safety Data management: Definitions and Standards for Expedited Reporting [Internet]. Genebra: International Conference on Harmonisation of Technical Requirements ror Registration of Pharmaceuticals for Human Use; 1994 [cited 2012 Oct 15]. E2A. Available at: http://www.ich.org/fileadmin/Public_Web_Site/ICH_Products/Guidelines/Efficacy/E2A/Step4/E2A_Guideline.pdf.

[3] NIC/NIH NCI Common Terminology Criteria for Adverse Events (CTCAE) [Internet]. Available at: http://evs.nci.nih.gov/ftp1/CTCAE/About.html.

[4] UMC/WHO The use of the WHO-UMC system for standardised case causality assessment [Internet]. Uppsala:The Uppsala Monitoring Centre. Available at: http://www.who-umc.org/Graphics/24734.pdf.
